# Effects of combined application of nitrogen fertilizer and biochar on the nitrification and ammonia oxidizers in an intensive vegetable soil

**DOI:** 10.1186/s13568-017-0498-7

**Published:** 2017-11-07

**Authors:** Qing-Fang Bi, Qiu-Hui Chen, Xiao-Ru Yang, Hu Li, Bang-Xiao Zheng, Wei-Wei Zhou, Xiao-Xia Liu, Pei-Bin Dai, Ke-Jie Li, Xian-Yong Lin

**Affiliations:** 1 Key Laboratory of Subtropical Soil Science and Plant Nutrition of Zhejiang Province, College of Environmental & Resource Sciences, Zhejiang University, Hangzhou, 310058 China; 2 MOE Key Laboratory of Environment Remediation and Ecological Health, College of Environmental & Resource Sciences, Zhejiang University, Hangzhou, 310058 China; 3 Key Lab of Urban Environment and Health, Institute of Urban Environment, Chinese Academy of Sciences, Xiamen, 361021 China; 4 Nanjing Institute of Environmental Sciences, Ministry of Environmental Protection, Nanjing, 210042 China; 5 Zhejiang Agricultural Technology Extension Center, Hangzhou, 310020 China; 6 Department of Applied Engineering, Zhejiang Economic and Trade Polytechnic, Hangzhou, 310018 China

**Keywords:** Nitrification, Ammonia-oxidizing community, Biochar, Vegetable soil

## Abstract

Soil amended with single biochar or nitrogen (N) fertilizer has frequently been reported to alter soil nitrification process due to its impact on soil properties. However, little is known about the dynamic response of nitrification and ammonia-oxidizers to the combined application of biochar and N fertilizer in intensive vegetable soil. In this study, an incubation experiment was designed to evaluate the effects of biochar and N fertilizer application on soil nitrification, abundance and community shifts of ammonia-oxidizing bacteria (AOB) and ammonia oxidizing archaea (AOA) in Hangzhou greenhouse vegetable soil. Results showed that single application of biochar had no significant effect on soil net nitrification rates and ammonia-oxidizers. Conversely, the application of only N fertilizer and N fertilizer + biochar significantly increased net nitrification rate and the abundance of AOB rather than AOA, and only AOB abundance was significantly correlated with soil net nitrification rate. Moreover, the combined application of N fertilizer and biochar had greater effect on AOB communities than that of the only N fertilizers, and the relative abundance of 156 bp T-RF (*Nitrosospira* cluster 3c) decreased but 60 bp T-RF (*Nitrosospira* cluster 3a and cluster 0) increased to become a single predominant group. Phylogenetic analysis indicated that all the AOB sequences were grouped into *Nitrosospira* cluster, and most of AOA sequences were clustered within group 1.1b. We concluded that soil nitrification was stimulated by the combined application of N fertilizer and biochar via enhancing the abundance and shifting the community composition of AOB rather than AOA in intensive vegetable soil.

**Electronic supplementary material:**

The online version of this article (10.1186/s13568-017-0498-7) contains supplementary material, which is available to authorized users.

## Introduction

Biochar, a carbon-rich product, was derived from the pyrolysis carbonization organic matter under anoxic or hypoxic and relatively low temperature conditions (≤ 700 °C) (Lehmann and Joseph 2015). Biochar with its potential agronomic benefits has been largely certified to exhibit strong improvement on soil quality (Lehmann 2007; Lehmann et al. 2006, 2011). To be specific, studies have indicated that adding biochar into soil could enhance nutrient availability and sequester carbon, increase soil pH and cation-exchange capacity as a soil conditioner, and also alter soil microbial populations resulting in impacting on nutrient cycling (Lehmann et al. 2011). For these reasons, biochar has been increasingly evaluated as a soil amendment to improve soil fertility generating higher productivity. Moreover, biochar play an important role in soil nitrogen (N) cycle via reducing inorganic-N leaching and N_2_O emission (Pan et al. 2017; Singh et al. 2010; Spokas and Reicosky 2009; Xu et al. 2014), increasing biological N fixation (Rondon et al. 2007) and enhancing N availability for crops (Zheng et al. 2013). Therefore, biochar may impact the process of soil nitrification.

Nitrification is a central process in the nitrogen cycle, by which microorganisms oxidize ammonium (NH_4_^+^) to generate nitrate (NO_3_^−^), making soil nitrogen available for crop growth (Kowalchuk and Stephen 2001). The rate-limiting step of nitrification is the oxidation of NH_4_^+^, which is driven by ammonia-oxidizing bacteria (AOB) and ammonia oxidizing archaea (AOA). Even though both AOB and AOA have been demonstrated as key drivers in ammonia oxidation in agricultural soil (Jin et al. 2010; Li and Gu 2013), their functional importance differs from various environmental conditions (He et al. 2007; Leininger et al. 2006).

Moreover, many studies have shown that biochar addition significantly accelerated soil nitrification and improved the amount of soil ammonia-oxidizing microorganisms (Nelissen et al. 2012; Song et al. 2013). In forest soil, the abundance of AOB and nitrification rate have been found to increase with the charcoal addition (DeLuca et al. 2006; Ball et al. 2010). This is explained by the biochar adsorption of nitrification-inhibiting compounds such as terpenes and phenols (Ball et al. 2010). Contrarily, some researches have shown that biochar addition had significant inhibiting effect on nitrification, which was attributed to the agricultural system with high nitrification rate (DeLuca et al. 2006) or the presence of nitrification-inhibiting compound (α-pinene) in the biochar (Clough et al. 2010). Given that the critical role of AOA and AOB in soil nitrification process, the effect of biochar in agricultural situations might be indirectly or partially via its impact on the ammonia-oxidizing community itself.

Compared with cereal production, more intensive cropping rotations and frequent irrigation, and much larger input of nutrients are always performed in the greenhouse vegetable system in China (Shen et al. 2010). That could lead to a series of problems such as soil acidification, salinization, hardening, and nutrient imbalance, causing soil degradation and yield reduction. Moreover, annual N fertilizer inputs are 3–4 times greater in the greenhouse vegetable system than that in the non-vegetable system (Ju et al. 2006), whereas the nitrogen use efficiency was very low in the intensive vegetable soil (He et al. 2006). Therefore, those problems are becoming a serious challenge to establish the sustainable intensive vegetable agricultural. As mentioned before, biochar with special physical and chemical properties can be used as a soil amendment, and has significant effects on alleviating soil acidification, improving soil structure, increasing soil available nutrients and vegetable yield (Chan et al. 2007). However, to our knowledge, little is known about biochar and N fertilizer interaction on nitrification and ammonia-oxidizing microbial community in the intensive vegetable soil. Therefore, we performed an incubation experiment to unravel the dynamic response of nitrification and ammonia oxidizers to the single application N fertilizer (urea and (NH_4_)_2_SO_4_) or biochar and the combined application of N fertilizer and biochar across a greenhouse vegetable soil. To differentiate the role of AOA and AOB, quantitative real-time PCR (qPCR) and terminal restriction fragment length polymorphism (T-RFLP) combined with clone libraries were used to determine the abundance and structure of ammonia-oxidizing microbial communities.

## Materials and methods

### Soil description and soil sampling

Soil sample was collected from a vegetable greenhouse in the urban–rural transitional area (30°17′ N, 120°13′ E) of Hangzhou City, China. Vegetables have been cultivated intensively for 30–40 years at this site (Chen et al. 2008). The soil was sandy loam (clay 6.2%, silt 33.7%, sand 60.1%) (Chen et al. 2015) with 3.1 g kg^−1^ total N (TN), 27.6 g kg^−1^ organic matter (OM) and pH value of 7.0. The collected soil samples from the top layer (0–15 cm) without any debris were grounded to pass through a 2-mm sieve after air-dried. A part of soil samples was used to measure the chemical properties, and the remainder were preserved for laboratory incubation experiment.

### Characterization of biochar

Biochar used in this experiment was produced from rice straw, which was carbonized under hypoxic condition at 600 °C. The biochar had a pH of 10.2, a total carbon (C) content of 53.7%, a TN content of 1.2%, a total hydrogen (H) content of 1.2%, a H:C ratio of 0.3, a C:N ratio of 53.5%, a 35.1% ash content.

### Experimental design

To revive soil microbial activity, the air-dried soil was pre-incubated at 25 °C and 60% of water-holding capacity (WHC) for 2 weeks. The experiment was conducted in 100 mL plastic jars containing 50 g soil with six treatments including control, urea, (NH_4_)_2_SO_4_, 2% biochar, 2% biochar + urea, 2% biochar + (NH_4_)_2_SO_4_, and each treatment was replicated for three times. The amount of 2% biochar added to soil was 2 mg kg^−1^ soil, which was calculated corresponding to a nitrogen addition of 200 mg N kg^−1^ soil. All these jars were then incubated inside incubator at 25 °C for 48 days. During the incubation period, soil moisture contents were kept constant at 65% WHC by adding deionized water based on the weighing method. The destructive sampling was performed during incubation period of 0, 1, 3, 7, 14, 21, 28, 35, 42 and 48 days.

### Soil property analysis

Soil pH was measured at a soil solution ratio of 1:5 (w/v) with a pH meter. Soil organic matter was determined by external-heat potassium dichromate oxidation-colorimetric method (Nelson and Sommers 1982). TN contents were measured by the Kjeldahl method. Grain size distribution of the soil samples was measured by a Mastersizer 2000 Laser Grainsize (Mastersizer 2000 Laser Grainsize, Malvern Instruments, Worcestershire, UK). Soil ammonium N (NH_4_^+^–N) and nitrate N (NO_3_^−^–N) were extracted by 2 mol L^−1^ KCl solution (soil/KCl, 1:5) and measured by a flow injection analyzer (FLA sta 5000 Analyzer, Foss, Denmark). Soil NH_4_^+^–N and NO_3_^−^–N contents at day 0 were determined using the preincubation soil. And the net nitrification rate (*n*) was calculated following the Persson and Wirén (1995) equation:

$$ n({\text{mg N kg}}^{ - 1} {\text{soil day}}^{ - 1} ) = \frac{{\left( {{\text{NO}}_{3}^{ - } - {\text{N}}} \right)_{t2} - \left( {{\text{NO}}_{3}^{ - } - {\text{N}}} \right)_{t1} }}{t} $$

Where *t* is the number of days between two sampling time (*t*2 and *t*1, day), and (NO_3_^−^–N)_*t*1_ and (NO_3_^−^–N)_*t*2_ are the nitrate concentrations at time 1 and time 2, respectively.

### Soil DNA extraction and quantitative real-time PCR of *amo*A genes

Soil DNA was extracted from ~ 0.5 g frozen samples using a FastDNA SPIN Kit for soil (Bio101, Vista, CA) according to the manufacturer’s protocol. The DNA was stored at − 20 °C for the molecular analyses described below.

The abundances of AOA and AOB were determined via qPCR on a Bio-Rad CFX 1000 real-time PCR machine. Two primer pairs were used for detecting the AOA and AOB (Additional file 1: Table S1). Each PCR reaction was performed in a 20-μL mixture containing 1 μL of tenfold diluted DNA, 0.5 μM of each primer and 10 μL of SYBR Premix EX Taq™, following with PCR protocol: initial denaturation at 95 °C for 3 min, 40 cycles of 95 °C for 10 s, 55 °C for 30 s and 72 °C for 40 s. Serial dilutions of linearized plasmids containing cloned *amoA* genes were conducted to make calibration curves. Only one peak at a melting temperature (Tm) was detected. Only the standard curves with PCR efficiencies of 90–110% and correlation coefficients > 0.99 were employed in this study.

### T-RFLP of *amo*A genes for ammonia oxidizers

For analysis of the ammonia oxidizers community, T-RFLP analysis was performed on DNA extracted from soil samples of all treatments at day 3. The same primers used in the qPCR with forward primer labeled with 6-FAM (6-carboxyfluorescein) were used in the T-RFLP analyses (Additional file 1: Table S1). The AOA and AOB samples were digested with restriction enzymes by HpyCH4V (NEB) and *Msp*Ι (NEB), respectively. Fragment size was carried out with an ABI PRISM 3030 × L genetic analyzer (Applied Biosystems, Warrington, UK). T-RFs (terminal restriction fragments) with sizes longer than 50 bp and percentages higher than 1% were kept for cluster analysis and the rest fragments were discarded.

### Cloning and sequencing

To identify the main T-RFs, the AOB and AOA clone libraries from the control soil were constructed with the same primers used in the qPCR analysis. Following manufacturer’s instructions, clones were generated by a TOPO® TA Cloning kit (Invitrogen, Carlsbad, CA). And the T-RFLPs were transformed into numerical data using ABI 3730 × L DNA analyzer (Applied Biosystems). Phylogenetic analyses were conducted with MEGA software (Tamura et al. 2013). The sequences were performed using the BLAST program in the GenBank database. Nucleotide sequences of *amoA* genes for the clone libraries in this study have deposited in GenBank under the accession numbers MF616026–MF616122.

### Statistical analysis

Correlation, variance analyses (ANOVA) and the multiple stepwise linear regression were performed by IBM SPSS Statistics version 21.0. Principal component analysis (PCA) were performed using R studio with VEGAN package. All figures were generated using OriginPro 8.5.

## Results

### Dynamics of NH_4_^+^–N and NO_3_^−^–N concentrations

**Fig. 1:**
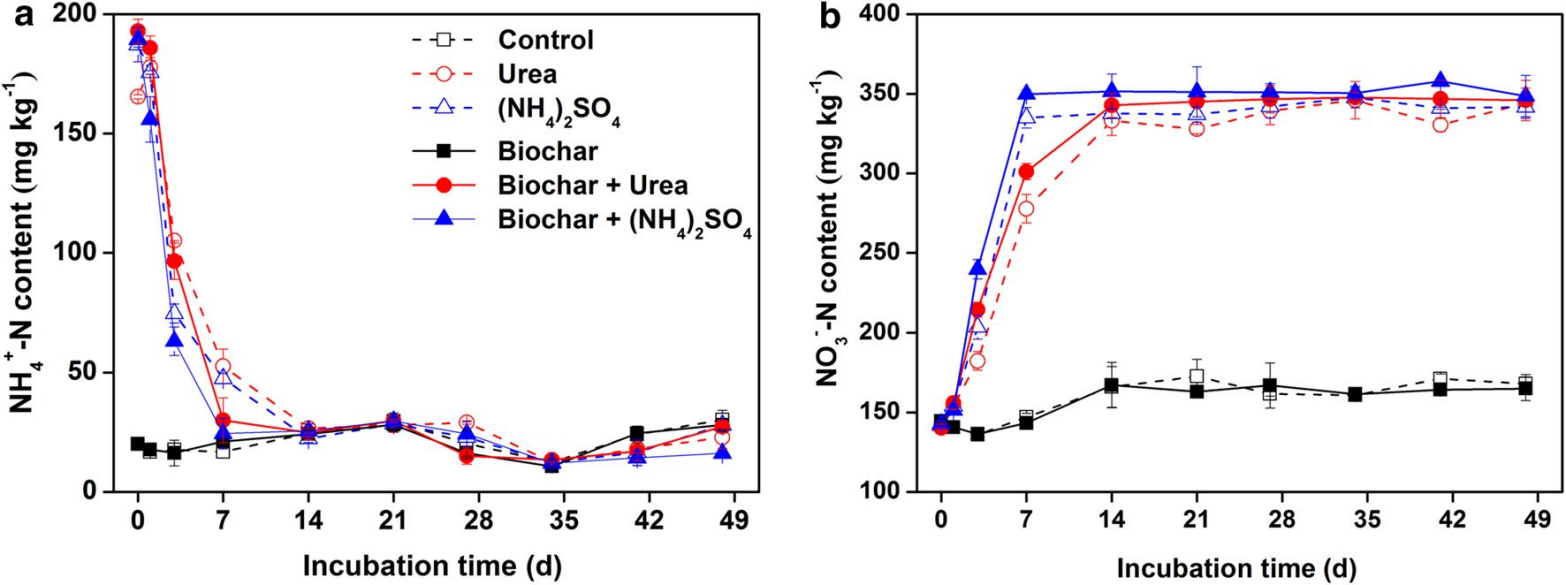
Dynamics of NH_4_^+^–N (**a**) and NO_3_^−^–N (**b**) contents after the application of biochar and nitrogen fertilizer in the greenhouse vegetable soil

No significant differences of NH_4_^+^–N and NO_3_^−^–N concentrations were found between only biochar amended and control treatments (Fig. 1). The urea and (NH_4_)_2_SO_4_ treatments showed an immediate NH_4_^+^ release with the highest NH_4_^+^–N concentrations (192.9 and 177.7 mg kg^−1^ in the urea and (NH_4_)_2_SO_4_ treatments, respectively) at day 1, and dramatically declined, but was no significant difference compared to the control after incubation of 14 days (Fig. 1a). Whereas the concentrations of NO_3_^−^–N increased rapidly and then kept stable (Fig. 1b). With the combination of biochar and nitrogen fertilizer addition, the rate of decrease in the NH_4_^+^–N concentration was higher than the single application of N fertilizer treatments before day 7, and NO_3_^−^–N concentration also showed greater increase.

**Fig. 2:**
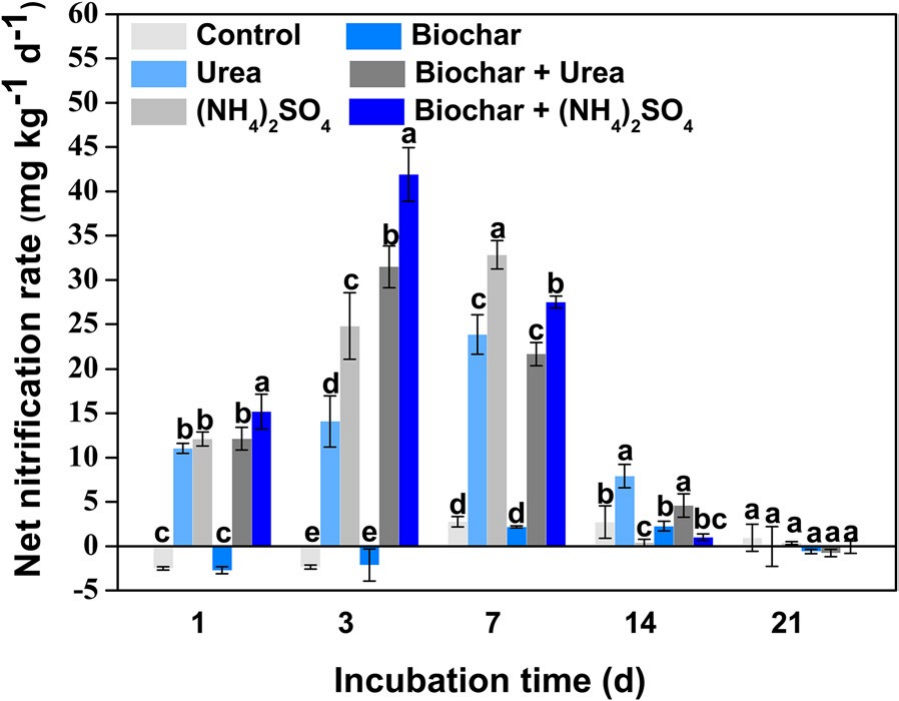
Dynamics of net nitrification rate after the application of biochar and nitrogen fertilizer in the greenhouse vegetable soil

For the net nitrification rate, there was no significant difference between the treatment with single application of biochar and control. Whereas the net nitrification rate significantly increased in the nitrogen fertilizer treatments (Fig. 2) (*p* < 0.05), which were greater in the (NH_4_)_2_SO_4_ treatments (24.8 ± 3.8 and 32.8 ± 1.6 mg kg^−1^ day^−1^ at day 3 and day 7, respectively) than in the urea treatments (14.1 ± 2.9 and 23.8 ± 2.2 mg kg^−1^ day^−1^ at day 3 and day 7, respectively). Additionally, for the biochar + N fertilizer treatments, the net nitrification rates were significantly higher than the single application of nitrogen fertilizers at day 3 (*p* < 0.05). Moreover, the net nitrification rate in the biochar + (NH_4_)_2_SO_4_ treatment (41.9 ± 2.4 mg kg^−1^ day^−1^) was significantly higher in comparison with the biochar +urea treatment (31.5 ± 3.0 mg kg^−1^ day^−1^). These results indicated that the combined application of nitrogen fertilizer and biochar could enhance the nitrification of vegetable soil.

### Abundance of ammonia oxidizers

**Fig. 3:**
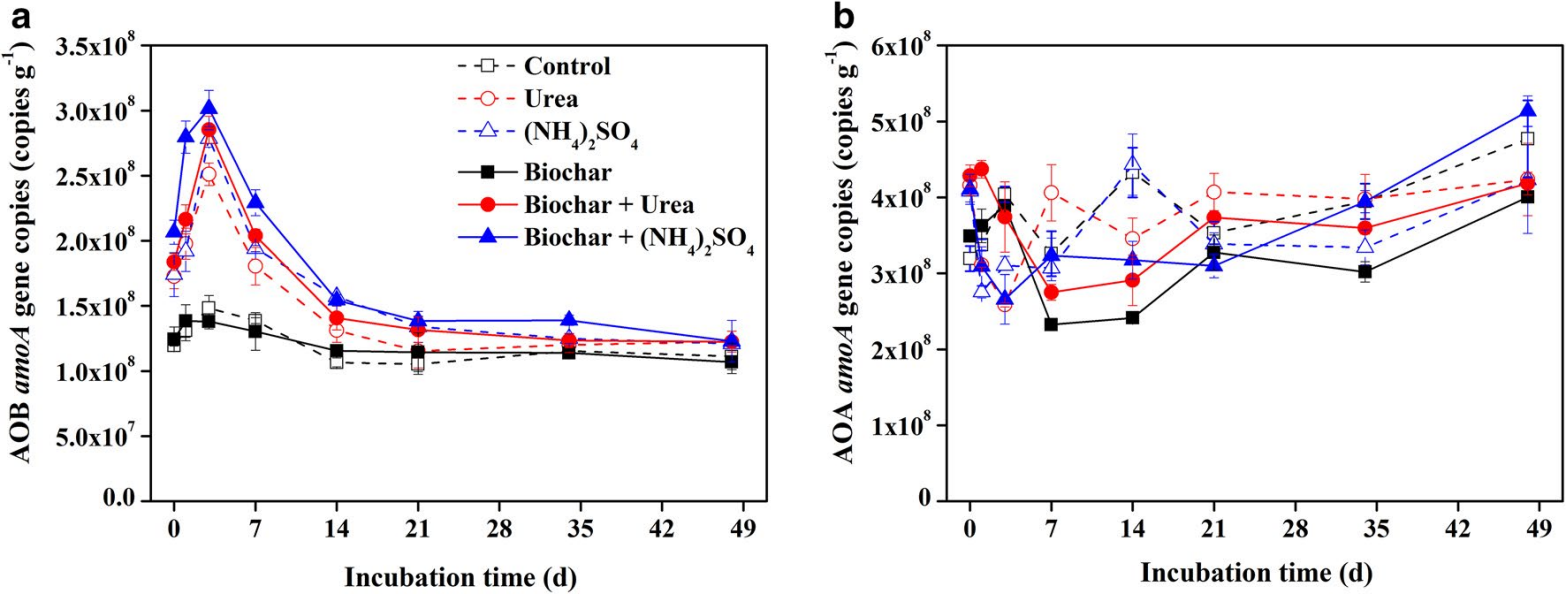
Dynamics of AOA (**a**) and AOB (**b**) *amo*A gene copies after the application of biochar and nitrogen fertilizer in the greenhouse vegetable soil

During the incubation, the abundance of *amoA* in all treatments ranged from 2.3 × 10^8^ to 5.1 × 10^8^ copies g^−1^ dry soil and 1.0 × 10^8^ to 3.0 × 10^8^ copies g^−1^ dry soil for AOA and AOB, respectively (Fig. 3). In this vegetable soil, the abundance of AOA was higher than AOB and the ratios of AOA to AOB ranged from 1.0 to 3.8. During the incubation, there was no significant difference on the AOB abundance between the only biochar treatment and the control. More interestingly, the AOB *amoA* gene abundances in all the N fertilizer amended treatments increased significantly (*p* < 0.05), which reached up from 1.6 to 2 times than the control at day 3. Furthermore, the AOB *amoA* gene abundances in the biochar + N fertilizer treatments were higher than that in the only N fertilizer treatments. Results also showed that the AOB *amoA* gene abundance was always higher in the (NH_4_)_2_SO_4_ treatments than that in the urea treatments (Fig. 3a). However, the application of biochar and nitrogen fertilizer had no effects on AOA *amo*A gene abundance (Fig. 3b).

### Community compositions of AOA and AOB

**Fig. 4:**
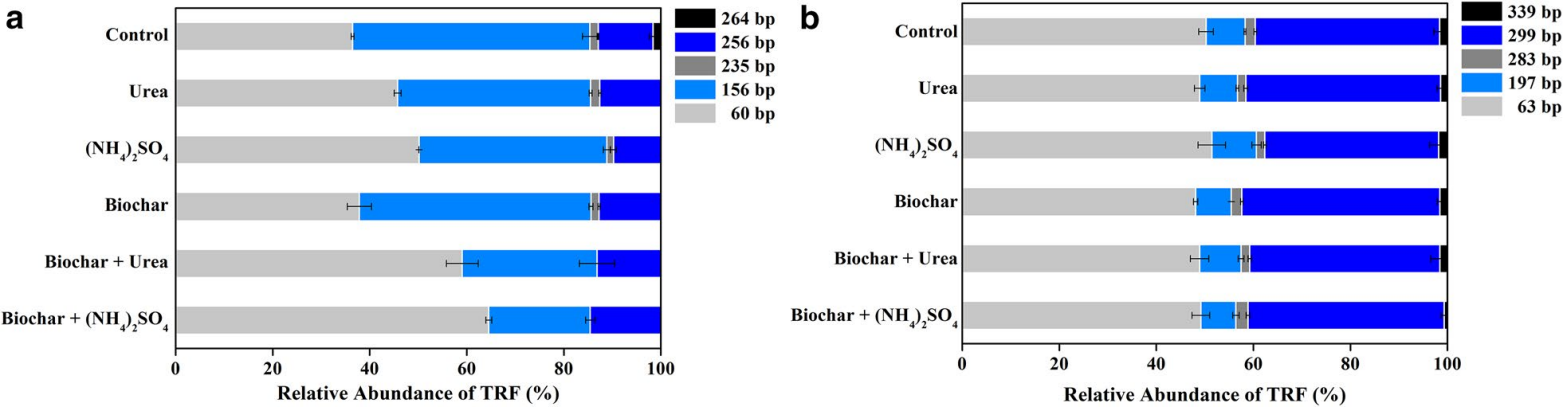
Relative abundance (**a**) and principle component analysis (**b**) of the ammonia oxidizing bacterial T-RFs

As shown in the Fig. 4, the application of different fertilizers had a great impact on AOB community compositions, but only slight variations were observed in AOA. The predominant T-RFs in AOB community were 60 and 156 bp, which accounted for above 80% of the total community (Fig. 4a). Except for 264 bp T-RF, only biochar treatment showed no difference from the control on the AOB T-RFLP profiles. With the addition of N fertilizer, the relative abundance of 60 bp T-RF increased significantly, but 156 bp T-RF decreased (*p* < 0.05). Moreover, the relative abundances of 60 bp T-RF were higher in the biochar + N fertilizer treatments than those in the only N fertilizer treatments. Additionally, the 235 bp T-RF was not detected in the biochar + N fertilizer treatments. PCA analysis of the AOB T-RFLP profiles further revealed that the two axes explained 84.7%, and the AOB community composition was significantly influenced by biochar and N fertilizer (Additional file 1: Figure S1). Results showed that both the biochar and N fertilizer treatments were clearly separated from the control, whereas only biochar and only N fertilizer treatments and the biochar + N fertilizer treatments were clustered together. Moreover, the Shannon and Simpson index are normally used to characterize species diversity in a community. For AOB community, both the Shannon and Simpson index decreased significantly in the only (NH_4_)_2_SO_4_ and biochar + N fertilizer treatments when compared with the control which were significantly reduced with the biochar and N fertilizer addition (*p* < 0.05) (Table 1). Overall, our results demonstrated that the AOB community diversity in treatments with combined application of biochar and N fertilizer shifted significantly.

**Table 1:** The Shannon index (H) and Simpson index (D) of AOB and AOA community structure diversity

	AOB		AOA	
	H	D	H	D
Control	1.099a	0.614a	1.063a	0.596a
Urea	1.060ab	0.616a	1.043ab	0.593a
(NH_4_)_2_SO_4_	0.999c	0.589b	1.070a	0.597a
Biochar	1.049b	0.611a	1.058a	0.596a
Biochar +Urea	0.932d	0.555c	1.067a	0.599a
Biochar + (NH_4_)_2_SO_4_	0.890d	0.518d	1.005b	0.583a

Different letters indicate significance at *p* < 0.05

### Phylogeny of AOA and AOB

**Fig. 5:**
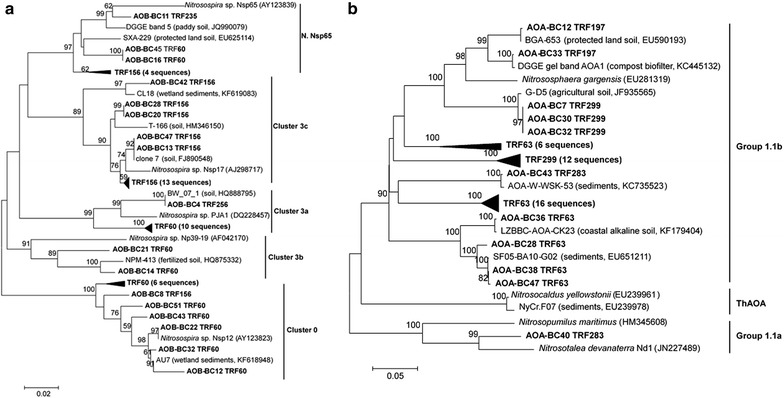
Neighbor-joining phylogenetic tree of **a** bacterial *amo*A sequences and **b** archaeal *amo*A sequences retrieved from the vegetable soil. Sequences from this study are shown in bold and described as clone name (accession number) T-RF size. Reference sequences are described as clone name (environment, accession number). Bootstrap values (> 50%) are indicated at branch points. The scale bar represents 5% estimated sequence divergence. The accession numbers in GenBank were MF616026–MF616122

The AOB phylogenetic tree showed that AOB sequences were divided into five distinct clusters including *Nitrosospira* cluster 3a, 3b, 3c, cluster 0 and *Nitrosospira* sp. Nsp65, affiliating to genus of *Nitrosospira* (Fig. 5). The 156 bp T-RF mainly belonged to *Nitrosospira* cluster 3c, the other 21% belonged to *Nitrosospira* sp. Nsp65 and cluster 0. And T-RF 60 bp distributed in various genera of *Nitrosospira*. The T-RFs of 235 and 256 bp were affiliated with *Nitrosospira* sp. Nsp65 and cluster 3a, respectively.

The AOA phylogenetic analysis indicated that AOA sequences of vegetable soil (98%) were highly homologous to *Nitrososphaera gargensis*, which belonged to group 1.1b, and only one sequence belonging to group 1.1a. The T-RFs of 197 bp, 299 bp and 63 bp were closely aligned with group 1.1b, and the 283 bp T-RF was affiliated with group 1.1a and group 1.1b.

## Discussion

In this study, no significant differences of NH_4_^+^–N, NO_3_^−^–N concentrations and net nitrification rates were observed between only biochar addition and control treatment (Fig. 1), indicating that biochar addition had little effect on the soil nitrification in the absence of N fertilizer. This result may be caused by the limited NH_4_^+^ in the collected soil sample, which are the substrate of hydrolysis by ammonia-oxidizing microorganisms, or the biochar with the carbon-rich but nutrient-poor characteristic (Alburquerque et al. 2013). As expected, soil NH_4_^+^–N concentrations significantly increased after the addition of N fertilizer, and sharply decreased to an equilibrium at day 7 or day 14 (Fig. 1a). In contrast, the NO_3_^−^–N concentrations showed a rapid increase and also reached up an equilibrium correspondingly (Fig. 1b). These results suggested that when adding the N fertilizer, the conversion from ammonium to nitrate for crop growth via nitrification was immediate and rapid. Additionally, the net nitrification rate reached up to 42 ± 3 mg kg^−1^ day^−1^ in this study (Fig. 2), indicating that the N fertilizer provided an obvious promotion in the nitrification of vegetable soil via ammonia-oxidizing microorganisms. Moreover, the NO_3_^−^–N concentrations and net nitrification rates in the biochar + N fertilizer treatments were observed to be higher than only N fertilizer treatments, which reached a significant higher level at day 3 and day 7 (*p* < 0.05), respectively, demonstrating that the combined application of N fertilizer and biochar had a synergistic effect on soil nitrification. Our findings were consistent with the previous study, which indicated that crop growth were simulated applied with biochar and mineral fertilizer (Asai et al. 2009; Schulz and Glaser 2012; Van Zwieten et al. 2010). As for biochar, the ability of promoting nitrification could be attributed to the adsorption of inhibiting substances of nitrification such as phenols and terpene in biochar (Ball et al. 2010; Berglund et al. 2004; DeLuca et al. 2006). Furthermore, biochar could significantly increase soil organic carbon, resulting in high ratios of carbon to nitrogen, which could enhance soil nitrification to improve the bioavailability of nitrogen (Clough et al. 2013). In addition, Zhao et al. (2013) found that the soil pH increased significantly after combined application of biochar and N fertilizer in an acid agricultural soil, which was also promoted with the increase of the amount of biochar.

The abundance of AOB increased significantly in N fertilizer treatments (*p* < 0.05), which was line with the higher net nitrification rate in these soils (Figs. 2 and 3a). Additionally, significantly positive correlation was also observed between net nitrification rate and AOB abundance rather than AOA abundance (*r* = 0.829**, *p* < 0.01) (Additional file 1: Table S2), permutational multivariate analyses also showed positive correlation in AOB abundance and the effects of different treatments and incubation time (Additional file 1: Table S3). This suggested that the increased abundance of AOB played a more direct positive role than AOA in the soil nitrification with N fertilizer, which was consistent with previous reports in natural and alkaline soil (Jia and Conrad 2009; Shen et al. 2008). Moreover, higher abundance of AOB was observed in N fertilizer + biochar treatments compared with only N fertilizer treatments, (Fig. 3a), indicating that combined application of N fertilizer with biochar could enhance the nitrification by increasing the abundance of AOB in this vegetable soil. However, the effects of biochar on ammonia-oxidizers were quite distinct from diverse soils. For example, with biochar addition in coastal saline soil, higher AOA abundance increased soil ammoxidation rate (Song et al. 2013). Whereas Prommer et al. (2014) found that biochar boosted both AOA and AOB abundances in agricultural soil, leading to the enhancement of soil potential nitrification rates. Overall, the increase in the amount of ammonia-oxidizing microorganisms associated with biochar addition may be due to the following four reasons. Firstly, large surface area and highly porous structure with water holding capacity and nutrient retention of biochar could provide resources for the specific metabolic needs of microorganisms (Steinbeiss et al. 2009). Secondly, biochar could improve living condition of biota by the increase of pH in acid soil (Ball et al. 2010). Thirdly, the source of carbon and nitrogen in biochar improve soil fertility. Finally, biochar might absorb the inhibiting substances such as polyphenols or tannins on nitrification (Ball et al. 2010; DeLuca et al. 2006). Nevertheless, some studies had reported that biochar addition showed no difference even a negative effect on soil nitrification (Clough et al. 2010; Spokas and Reicosky 2009). That may be due to the release of nitrification inhibitor such as ethylene and α-pinene via biochar to reduce soil ammonia-oxidizing microorganism activity (Berglund et al. 2004; DeLuca et al. 2006), which was different on the parent materials and conditions during the formation of biochar.

The T-RFLP analysis showed that the AOB community structures varied from different treatments (Fig. 5). The dominance of *Nitrosospira* cluster 3 (contributing to 62% of the sequences) in AOB indicated that *Nitrosospira* cluster 3 played an important role in nitrification (Shen et al. 2011), whereas *Nitrosomonas* was not detected in the greenhouse vegetable soil (Fig. 5b). This was consistent with a previous study that *Nitrosospira* cluster 3 also dominated in a long-term fertilization sandy loam soil (Chu et al. 2007). Our results also revealed that the community structure and diversity of AOB in vegetable soil were significantly altered by the combined application of biochar and N fertilizer rather than the single application of biochar (Fig. 4). Compared with the control, the relative abundance of 60 bp T-RF increased from 36.5 to 64.6% in the biochar + (NH_4_)_2_SO_4_ treatment, which belonged to *Nitrosospira* cluster 3a and cluster 0, suggesting that the combined application of biochar and N fertilizer stimulated microbial growth of the related cluster. However, 156 bp T-RF belonging to *Nitrosospira* cluster 3c showed a significant decrease. Dempster et al. (2011) also found AOB community shifts occurred in biochar + N fertilizer treatments but not in the single biochar added treatment. Moreover, the significant decrease of Shannon and Simpson index also reflected the reduction in community diversity of AOB with the combined addition of biochar and N fertilizer in this vegetable soil (Table 1). The dominant AOA was affiliated to group 1.1b containing 98% sequences. Although many studies have indicated that AOA are considered to be the primary driver of nitrification (Chen et al. 2008; He et al. 2007; Leininger et al. 2006), in our study, there was no discernable changes in the AOA community after the combined application of N fertilizer and biochar, indicating the AOB community was more sensitive to the fertilization practice in vegetable soil. In agreement with our findings, extensive research have observed an obvious promotion effect of fertilizer on AOB community rather than AOA (Ai et al. 2013; Fan et al. 2011; Xia et al. 2011). In general, the variance in ecological niches of AOB and AOB are caused by their dissimilar sensitivity to soil properties (Shen et al. 2008). As AOB are considered to mainly dominated in the neutral and “nutrient-rich” environment, whereas AOA are better to adapted to low pH and “nutrient-poor” environment (Schauss et al. 2009). Furthermore, the reason that the significant shift in AOB community occurred in this study, may be the increase of soil pH and nutrient contents after the biochar (pH = 10.2) and N fertilizer addition.

In conclusion, our results revealed that N fertilizer with the addition of biochar significantly stimulated soil nitrification and shifted the AOB abundance and community. T-RFLP of AOB indicated that the combined application of N fertilizer and biochar significantly increased the 60 bp T-RF (*Nitrosospira* cluster 3a and cluster 0) but decreased 156 bp T-RF (*Nitrosospira* cluster 3c). On the contrary, there were no visible changes in the AOA community compared to AOB. Moreover, the positive correlation between net nitrification rate and AOB abundance, indicating that AOB rather than AOA was the dominant ammonia oxidizer to drive soil nitrification in intensive vegetable soil. This has important implications that the combined utilization of N fertilizer and biochar enable to promote the nitrogen use efficiency.

## Additional file

**Additional file 1: Table S1.** Primers of AOA and AOB used for molecular analyses. **Table S2.** Pearson correlation between the abundance of AOA/AOB and net nitrification rate. **Table S3.** Permutational multivariate analyses for the effects of different treatment (Treat) and incubation time (Time) on the abundance of AOA and AOB. **Figure S1.** The principal coordinates analysis (PCA) of AOB T-RFs in vegetable soils treated with urea, (NH_4_)_2_SO_4_, biochar, biochar + urea, biochar + (NH_4_)_2_SO_4_ based on Bray-Curtis distance.

## Abbreviations

N: nitrogen
AOA: ammonia-oxidizing archaea
AOB: ammonia-oxidizing bacteria
qPCR: quantitative real-time PCR
T-RFLP: terminal restriction fragment length polymorphism
NH_4_^+^: ammonium
NO_3_^-^: nitrate
TN: total nitrogen
OM: organic matter
C: carbon
H: hydrogen
WHC: water-holding capacity
T-RFs: terminal restriction fragments
ANOVA: variance analyses
PCA: principal component analysis
