# Supplementary material for: Effects of combined application of nitrogen fertilizer and biochar on the nitrification and ammonia oxidizers in an intensive vegetable soil
Source: AMB Express. 2017 Nov 7;7:198. doi: 10.1186/s13568-017-0498-7 (PMC5676586; doi:10.1186/s13568-017-0498-7)
Supplement: Supplementary file 1 — Additional file 1: Table S1. Primers of AOA and AOB used for molecular analyses. Table S2. Pearson correlation between the abundance of AOA/AOB and net nitrification rate. Table S3. Permutational multivariate analyses for the effects of different treatment (Treat) and incubation time (Time) on the abundance of AOA and AOB. Figure S1. The principal coordinates analysis (PCA) of AOB T-RFs in vegetable soils treated with urea, (NH4)2SO4, biochar, biochar + urea, biochar + (NH4)2SO4 based on Bray-Curtis distance. [file 13568_2017_498_MOESM1_ESM.docx]

**Additional file**

Applied Microbiology and Biotechnology Express

Effects of combined application of nitrogen fertilizer and biochar on the nitrification and ammonia oxidizers in an intensive vegetable soil

Qing-Fang Bi ^1,3,#^, Qiu-Hui Chen ^4,#^, Xiao-Ru Yang ^3^, Hu Li ^3^, Bang-Xiao Zheng ^3^, Wei-Wei Zhou ^1^, Xiao-Xia Liu ^5^, Pei-Bin Dai ^6^, Ke-Jie Li ^2^, Xian-Yong Lin ^1,2^*

^1^ Key Laboratory of Subtropical Soil Science and Plant Nutrition of Zhejiang Province, College of Environmental & Resource Sciences, Zhejiang University, Hangzhou 310058, China

^2^ MOE Key Laboratory of Environment Remediation and Ecological Health, College of Environmental & Resource Sciences, Zhejiang University, Hangzhou 310058, China

^3^ Key Lab of Urban Environment and Health, Institute of Urban Environment, Chinese Academy of Sciences, Xiamen 361021, China

^4^ Nanjing Institute of Environmental Sciences, Ministry of Environmental Protection,

Nanjing 210042, China

^5^ Zhejiang Agricultural Technology Extension Center, Hangzhou 310020, China

^6^ Department of Applied Engineering, Zhejiang Economic and Trade Polytechnic, Hangzhou 310018, China

# Qing-Fang Bi and Qiu-Hui Chen contributed equally to this work.

*Correspondence. E-mail: [xylin@zju.edu.cn](mailto:xylin@zju.edu.cn); Tel: +86 571-88982476; Fax: +86 571-86971395

Table S1 Primers of AOA and AOB used for molecular analyses

| Target group | Primer | Sequence (5'-3') | | Length of amplicon (bp) | | Reference |
| --- | --- | --- | --- | --- | --- | --- |
| AOA | CrenamoA23f | | ATGGTCTGGCTWAGACG | | 635 | Nicol et al. (2008) |
|  | CrenamoA616r | | GCCATCCATCTGTATGTCCA | |  |  |
| AOB | amoA-1F | | GGGGTTTCTACTGGTGGT | | 491 | Rotthauwe et al. (1997) |
|  | amoA-2R | | CCCCTCKGSAAAGCCTTCTTC | |  |  |

Table S2 Pearson correlation between the abundance of AOA/AOB and net nitrification rate

| Item | AOA | AOB |  | Net nitrification rate | |  |
| --- | --- | --- | --- | --- | --- | --- |
| AOA | 1 | -0.227* |  | | -0.248* | |
| AOB | -0.227* | 1 |  | | 0.829** | |
| Net nitrification rate | -0.248* | 0.829** |  | | 1 | |

The correlation value is significant at *p* < 0.05 (*) or *p* < 0.01 (**) (two-tailed).

Table S3 Permutational multivariate analyses for the effects of different treatment (Treat) and incubation time (Time) on the abundance of AOA and AOB

|  | AOA | |  | AOB | |
| --- | --- | --- | --- | --- | --- |
|  | R^2a^ | *p*^b^ |  | R^2^ | *p* |
| Treat | 0.285 | 0.006 |  | 0.156 | <0.001** |
| Time | - | - |  | 0.382 | <0.001** |
| Treat×Time | - | - |  | 0.532 | <0.001** |

^a^ R^2^-value (effect size) shows the percentage of variation explained by the categories.
^b^ Significant differences are indicated at *p* < 0.05 (*) or *p* < 0.01 (**).





Figure S1 The principal coordinates analysis (PCA) of AOB T-RFs in vegetable soils treated with urea, (NH_4_)_2_SO_4_, biochar, biochar + urea, biochar + (NH_4_)_2_SO_4_ based on Bray-Curtis distance.

**References**

Nicol GW, Leininger S, Schleper C, Prosser JI (2008) The influence of soil pH on the diversity, abundance and transcriptional activity of ammonia oxidizing archaea and bacteria. Environ Microbiol 10:2966–2978

Rotthauwe JH, Witzel KP, Liesack W (1997) The ammonia monooxygenase structural gene *amoA* as a functional marker: molecular fine-scale analysis of natural ammonia-oxidizing populations. Appl Environ Microbiol 63:4704-4712
